# The Association of Sleep Duration With Vision Impairment in Middle-Aged and Elderly Adults: Evidence From the China Health and Retirement Longitudinal Study

**DOI:** 10.3389/fmed.2021.778117

**Published:** 2021-12-24

**Authors:** Mengsha Sun, Qiyu Bo, Bing Lu, Xiaodong Sun, Minwen Zhou

**Affiliations:** ^1^Department of Ophthalmology, Shanghai East Hospital, Tongji University School of Medicine, Shanghai, China; ^2^Department of Ophthalmology, Shanghai General Hospital, Shanghai First People's Hospital, Shanghai Jiao Tong University School of Medicine, Shanghai, China; ^3^National Clinical Research Center for Eye Diseases, Shanghai, China; ^4^Shanghai Key Laboratory of Fundus Diseases, Shanghai, China

**Keywords:** vision impairment, sleep duration, association, adults, China

## Abstract

**Objective:** This study aims to investigate the association of sleep duration with vision impairment (VI) in middle-aged and elderly adults.

**Methods:** This cross-sectional study used the data from the baseline survey of the China Health and Retirement Longitudinal Study (CHARLS) 2011–2012, a national survey of adults aged 45 years or older. Weighted multilevel logistic regression models were used to evaluate the association between self-reported sleep duration and VI.

**Results:** Of the 13,959 survey respondents, a total of 4,776 (34.2%) reported VI. The prevalence of short (≤6 h/night) and long (>8 h/night) sleep durations was higher among respondents with VI than those without VI (*P* < 0.001). Multilevel logistic regression models showed that compared with a sleep duration of 6–8 h/night, a sleep duration of ≤6 h/night was associated with a 1.45-fold [95% confidence interval (CI) = 1.34–1.56] higher VI risk, and a sleep duration of >8 h/night was associated with a 1.18-fold (95% CI = 1.03–1.34) higher VI risk, after adjusting for sociodemographic data, lifestyle factors, and health conditions. Vision impairment was associated with short sleep duration in respondents from all age or gender categories. However, VI was associated with long sleep duration in respondents from the elderly or female categories. The association between VI and long sleep duration disappeared in respondents of middle-aged or male categories.

**Conclusions:** The potential impact of sleep on the risk of visual functions requires further attention. A more comprehensive and integrated health care and rehabilitation system covering vision and sleep is also needed.

## Introduction

Vision impairment (VI) is a major global public health problem. According to a published report in Lancet Global Health, the number of people affected by VI has increased substantially, a situation attributable to population growth and aging (1). The combination of cataract and uncorrected refractive error contributed to 55% of the blindness and 77% of the VI in adults aged 50 years and older in 2015 (1). In China, the prevalence of low bilateral vision was 5.1%, and the prevalence of blindness was 1.0%, based on the World Health Organization (WHO) best corrected visual acuity (BCVA) criteria in the Taizhou Eye Study (2). Further analysis revealed that VI was associated with an increased rate of cognitive impairment and depression and a lowered quality of life (3–5). A few studies based on the US population have concluded that older adults with VI also experienced increased sleep disturbance (6, 7).

In terms of VI, sleep is one of the aspects of functioning that may be underestimated because attention is focused on establishing visual functions. Sleep is vital for optimal physical and mental health, and growing evidence now supports an association of both short- and long-duration sleep with adverse health outcomes (8–10). Several epidemiologic studies have reported an increased risk of all-cause mortality, as well as stroke and cardiovascular disease mortality, among those with self-reported short or long sleep durations (11–13). Recent findings have also uncovered differences in sleep health based on racial/ethnic and socioeconomic position differences. The underlying causes of disparities in sleep health are only beginning to emerge (14).

Recent studies have reported a statistically significant association between sleep disorders and ocular diseases, such as glaucoma, optic nerve dysfunction, and dry eye syndrome (15–17). However, sleep data have not yet been considered an essential element in many epidemiologic studies of VI. To our knowledge, few large-scale population-based studies have examined the potential association between sleep duration and VI in an Asian population. The aim of the present study was to evaluate the associations between sleep duration and the prevalence of VI in a nationally representative survey of the Chinese adult population aged 45 years or older, using data from the 2011 baseline survey of the China Health and Retirement Longitudinal Study (CHARLS).

## Materials and Methods

### Data and Study Sampling

The CHARLS is a nationally representative survey of Chinese adults aged 45 years or older and their spouses. The national baseline survey for CHARLS was conducted between June 2011 and March 2012 and included 17,708 respondents in 150 counties or districts and 450 villages or urban communities throughout China (18). The CHARLS protocol followed the tenets of the Declaration of Helsinki (19) and received ethical approval from the institutional review board of Peking University (18). Informed consent was obtained from all participants when CHARLS was administered, consistent with the Strengthening the Reporting of Observational Studies in Epidemiology (STROBE) reporting guidelines (20).

### Vision Impairment

Vision consisted of both distance vision and near vision. Distance vision was assessed by asking the participants: “How good is your eyesight for seeing things at a distance, like recognizing a friend from across the street (with glasses or corrective lenses if you wear them)?” Near vision was assessed by asking the participants: “How good is your eyesight for seeing things up close, like reading ordinary newspaper print (with glasses or corrective lenses if you wear them)?” Responses to these two questions were divided into a five-point scale (excellent, very good, good, fair, and poor). A response of “poor” in distance or near vision was categorized as VI, while a response of “fair” or above in distance and near vision was categorized as no VI (21).

### Sleep Duration

Sleep duration was assessed by asking participants the following question: “During the past month, how many hours of actual sleep did you get at night (average hours for one night)? (This may be shorter than the number of hours you spend in bed).” The participant responses for sleep duration were categorized into three groups: (1) those reporting short sleep duration (≤6 h), (2) those reporting long sleep duration (>8 h), and (3) those reporting 6–8 h of sleep (used as the reference group) (22, 23).

### Covariates

Several potential confounding variables were taken into account, including participants' sociodemographic data, lifestyle factors, and health conditions. The sociodemographic data and lifestyle factors included in the analysis were as follows: age, sex (male or female), marital status (married or partnered vs. otherwise), smoking status (yes or no), drinking status (more than once a month, less than once a month, or none), and educational attainment (less than elementary school, elementary school, middle school, or high school or above).

Health conditions included in the analysis were hypertension (24, 25), dyslipidemia (26), diabetes (27), and chronic kidney disease (28–30), assessed by a combination of physical examination and self-report according to reference definition. In CHARLS, blood samples were collected and blood pressure was measured using a standard protocol by medically trained staff from the China Center for Disease Control and Prevention (31).

### Statistical Analysis

Because CHARLS has a complex sampling design, sampling weights were considered in the analyses. Continuous variables were presented using mean ± SD. Categorical variables were presented using numbers. Characteristics of the study sample were compared using the χ^2^-test for categorical variables and an independent-sample *t*-test for continuous variables. The subjects were divided into two groups according to gender or the median age: middle age and elderly. The prevalence of VI was calculated based on the complex survey design and non-response rate.

The PROC SURVEYFREQ procedure was used to calculate the overall age and gender-specific prevalence of VI using the inverse probability weighting method. Weighted multilevel logistic regression models were used to evaluate the odds ratio (OR) at a 95% confidence interval (CI). They modeled the association between self-reported sleep duration and VI, adjusting for sociodemographic data (age, sex), lifestyle factors (marital status, smoking status, drinking status, and education), and health conditions (hypertension, dyslipidemia, diabetes, and chronic kidney disease). Statistical analysis was carried out using the Statistical Analysis System (SAS) (version 9.4, SAS Institute, Cary, NC, USA) for Windows. All reported *P*-values were two-tailed, and values <0.05 were considered statistically significant.

## Results

After filtering, a total of 13,959 participants were deemed eligible for the current study. In total, 6,574 males and 7,385 females were included in the final analysis. The mean age was 59.97 (9.84) years. The characteristics of the included subjects in the CHARLS 2011–2012 are shown in Table 1. Of the study subjects, 4,776 reported VI, with a higher proportion of females than males reporting VI. Those who reported VI were older, less educated, and more likely to be unmarried or unpartnered. The subjects with VI were also more likely to have smoking history, hypertension, diabetes, and chronic kidney disease. Interestingly, sleep durations showed some relationships with VI. The prevalence of short (≤6 h/night) and long (>8 h/night) sleep durations was higher among respondents with VI than those without VI (*P* < 0.001).

**Table 1:** Demographic characteristics of middle-aged and elderly Chinese with and without suffering vision impairment (CHARLS 2011–2012).

**Variables**	**Vision impairment**	**No vision impairment**	***P***
**Total**	4,776	9,183	
**Age, year**	60.96 ± 9.87	59.09 ± 9.58	<0.001
**Sex**			<0.001
Male	1,980	4,594	
Female	2,796	4,589	
**Marital status**			<0.001
Married or partnered	4,055	8,191	
Otherwise	721	992	
**Smoking status**			0.002
Yes	1,760	3,633	
No	3,016	5,550	
**Drinking status**			0.126
Drink more than once a month	1,084	2,376	
Drink but less than once a month	313	765	
None	3,379	6,042	
**Education**			<0.001
Less than elementary school	3,591	5,697	
Elementary school	777	2,116	
Middle school	256	813	
High school or above	152	557	
**Hypertension**			<0.001
Yes	1,994	3,339	
No	2,782	5,844	
**Dyslipidemia**			0.060
Yes	1,624	2,977	
No	3,152	6,206	
**Diabetes**			<0.001
Yes	727	1,115	
No	4,049	8,068	
**Chronic kidney disease**			<0.001
Yes	407	495	
No	4,369	8,688	
**Sleep duration**			<0.001
≤6 h	2,718	4,252	
6–8 h	1,646	4,138	
>8 h	412	793	

The weighted VI prevalences in respondents, by age and sex, based on sleep durations are shown in Table 2. The prevalence of VI among the study respondents was 35.83%. More specifically, the prevalence of VI was higher among respondents with short (40.78%) or long (35.33%) sleep durations than those with sleep durations of 6–8 h (26.10%).

**Table 2:** Vision impairment prevalence in middle-aged and elderly Chinese according to sleep durations (CHARLS 2011–2012).

		**Sleep duration groups (h/night)**			***P***
	**Overall**	**≤6 h**	**6–8 h**	**>8 h**	
**All**	*n* = 13,959	*n* = 6,970	*n* = 5,784	*n* = 1,205	
Vision impairment, *n* (%)	4,776 (35.83)	2,718 (40.78)	1,646 (26.10)	412 (35.33)	<0.001
**Age**					
≥45 and ≤ 58 years	*n* = 7,239	*n* = 3,315	*n* = 3,320	*n* = 604	
Vision impairment, *n* (%)	2,101 (31.08)	1,125 (35.78)	813 (23.60)	163 (25.80)	<0.001
>58 years	*n* = 6,720	*n* = 3,655	*n* = 2,464	*n* = 601	
Vision impairment, *n* (%)	2,675 (40.98)	1,593 (42.97)	833 (31.68)	249 (40.19)	<0.001
**Sex**					
Male	*n* = 6,384	*n* = 3,172	*n* = 2,849	*n* = 553	
Vision impairment, *n* (%)	1,980 (32.89)	1,097 (35.61)	721 (26.88)	162 (30.82)	<0.001
Female	*n* = 7,385	*n* = 3798	*n* = 2,935	*n* = 652	
Vision impairment, *n* (%)	2,796 (38.47)	1,621 (45.84)	925 (30.26)	250 (39.65)	<0.001

By age, the VI prevalence was higher in elderly respondents (40.98%) than in middle-aged respondents (31.08%). Separately, the VI prevalence was higher in elderly respondents with short (42.97%) or long (40.19%) sleep durations than with sleep durations of 6–8 h (31.68%). The VI prevalence was also higher in middle-aged respondents with short (35.78%) or long (25.80%) sleep durations than with sleep durations of 6–8 h/night (23.60%).

The VI prevalence was also higher in female respondents (38.47%) than in male respondents (32.89%). In the female participants, the VI prevalence was 45.84, 30.26, and 39.65% for subjects with sleep durations of ≤6, 6–8, and >8 h/night, respectively. In the male participants, the equivalent VI prevalence was 35.61, 26.88, and 30.82%, respectively. Overall, the VI prevalence was higher among respondents with short or long sleep durations than with normal sleep duration in all age and gender categories.

The associations between sleep duration and VI determined using multilevel logistic regression models are shown in Table 3. These associations had variations after covariate adjustments for age, sex, marital status, smoking status, drinking status, education, hypertension, dyslipidemia, diabetes, and chronic kidney disease. Even after adjusting for these factors, a sleep duration of ≤6 h/night was associated with a 1.45-fold (95% CI = 1.34–1.56) higher VI risk, while a sleep duration of >8 h/night was associated with a 1.18-fold (95% CI = 1.03–1.34) higher VI risk compared with 6–8 h/night of sleep.

**Table 3:** Associations between sleep duration and risk of vision impairment in middle-aged and elderly Chinese (CHARLS 2011–2012).

	**Sleep duration groups (h/night)**		
	**≤6 h**	**6–8 h**	**>8 h**
	**OR (95%CI)**	**OR (95%CI)**	**OR (95%CI)**
**All**	*n* = 6,970	*n* = 5,784	*n* = 1,205
Model 1	1.50 (1.39–1.61)^*^	1.00 (Ref.)	1.23 (1.07–1.40)^*^
Model 2	1.47 (1.36–1.58)^*^	1.00 (Ref.)	1.18 (1.03–1.35)^*^
Model 3	1.45 (1.34–1.56)^*^	1.00 (Ref.)	1.18 (1.03–1.34)^*^
**Age**			
≥45 and ≤58 years	*n* = 3,320	*n* = 3315	*n* = 604
Model 1	1.50 (1.35–1.68)^*^	1.00 (Ref.)	1.15 (0.95–1.41)
Model 2	1.47 (1.32–1.64)^*^	1.00 (Ref.)	1.12 (0.91–1.36)
Model 3	1.45 (1.30–1.62)^*^	1.00 (Ref.)	1.11 (0.91–1.35)
>58 years	*n* = 3,655	*n* = 2,464	*n* = 601
Model 1	1.46 (1.31–1.63)^*^	1.00 (Ref.)	1.35 (1.12–1.62)^*^
Model 2	1.45 (1.30–1.62)^*^	1.00 (Ref.)	1.29 (1.07–1.55)^*^
Model 3	1.44 (1.29–1.60)^*^	1.00 (Ref.)	1.30 (1.08–1.57)^*^
**Sex**			
Male	*n* = 3,172	*n* = 2,849	*n* = 553
Model 1	1.50 (1.34–1.67)^*^	1.00 (Ref.)	1.15 (0.94–1.41)
Model 2	1.47 (1.31–1.65)^*^	1.00 (Ref.)	1.11 (0.90–1.36)
Model 3	1.45 (1.29–1.62)^*^	1.00 (Ref.)	1.11 (0.90–1.36)
Female	*n* = 3,798	*n* = 2,935	*n* = 652
Model 1	1.49 (1.35–1.65)^*^	1.00 (Ref.)	1.29 (1.08–1.54)^*^
Model 2	1.46 (1.32–1.62)^*^	1.00 (Ref.)	1.23 (1.03–1.48) ^*^
Model 3	1.45 (1.30–1.60)^*^	1.00 (Ref.)	1.24 (1.03–1.48)^*^

*Model 1 adjusted for age and sex. Model 2 adjusted for covariates in model 1 plus marital status, smoking status, drinking status, and education. Model 3 adjusted for covariates in model 2 plus hypertension, dyslipidemia, diabetes, and chronic kidney disease*.

* *Significant*.

A stratified analysis of age categories revealed that short (OR = 1.44, 95% CI: 1.29–1.60) and long (OR = 1.30, 95% CI: 1.08–1.57) sleep durations were associated with VI in elderly respondents. However, in middle-aged respondents, an association was only detected between short sleep duration and VI (OR = 1.45, 95% CI: 1.30–1.62). The association between long sleep duration and VI (OR = 1.11, 95% CI = 0.91–1.35) was absent among the middle-aged respondents.

A stratified analysis of gender categories revealed that short (OR = 1.45, 95% CI: 1.30–1.60) and long (OR = 1.24, 95% CI: 1.03–1.48) sleep durations were associated with VI in female respondents. However, male respondents showed only the association between short sleep duration and VI (OR = 1.45, 95% CI: 1.29–1.62). The association between long sleep duration and VI (OR = 1.11, 95% CI = 0.90–1.36) was absent among the male respondents.

## Discussion

This study examined the association between sleep duration and VI in a nationally representative sample of older adults in China. The use of large-scale population-based data in this study revealed several findings. First, self-reported VI was highly prevalent among middle-aged and elderly adults in China. Second, short (≤6 h/night) and long (>8 h/night) sleep durations were significantly associated with VI risks after adjusting for internal and external factors. Our findings suggest that both short and long sleep durations could be predictors of VI.

The prevalence of VI in China was higher than that reported in the US or Asia (32). The rapidly aging Chinese population may account for this higher rate of VI. Chen et al. (33) reported that cataracts were the leading cause of bilateral or monocular VI among adults 50 years and older in the Binhu District of Wuxi City, China, followed by high myopic macular degeneration, age-related macular degeneration, and eye loss/atrophy. Bourne et al. (34) reported that the leading causes worldwide were cataracts for blindness and uncorrected refractive error for moderate and severe VI. Moreover, more women than men were blind or showed moderate and severe VI due to cataracts and macular degeneration worldwide (34). Our results also revealed a higher prevalence of VI in women than in men, in agreement with previous studies. Therefore, more targeted efforts are required to prevent low vision and blindness in high-risk groups.

The mechanism that regulates sleep duration has not been fully explained. A short sleep duration may be attributed to difficulty in falling asleep, sleep fragmentation, and early awakening. Long sleep duration may occur due to poor circadian entrainment, compensation for fragmented sleep, and lower sleep efficiency (10, 35). Sleep deterioration and related VI may also result from several diseases (36). In turn, VI may further increase mood disturbances and even worsen sleep disorders. Further, respondents with VI tend to exhibit a shortened photoperiod and reduced circadian entrainment (8). The decline in or loss of light perception may lead to circadian misalignment, further contributing to abnormal sleep durations. Therefore, ophthalmologists must recognize the possibility of sleep disturbance in subjects with VI and participate in effective sleep management.

In addition to the above biological and clinical reasons, social factors should not be underestimated. Certain socioeconomic factors (including country of origin, acculturation, education, and annual income) have been found to be significantly associated with both short and long sleep duration (37, 38). These relationships persisted when controlling for overall health status. On the other hand, socioeconomic status is known to be an important determinant of VI (39). As such, socioeconomic status may play a role in the relationship between sleep duration and VI.

In particular, the results from the multilevel logistic regression models indicated a more significant potential for VI with short sleep duration than with long sleep duration. This suggests that persons who sleep for a short duration may require greater amounts of sleep and that worse sleep conditions pose a greater risk of VI. Additional studies examining the causal relationship between sleep durations and VI are needed.

To our knowledge, this is the first large-scale population-based study to explore the associations between self-reported sleep duration and VI among Chinese persons. Self-reported sleep duration and the prevalence of VI are known to reflect ethnic differences (40, 41). Therefore, investigation of the association between VI and sleep duration is essential among older adults in China. This type of research would be more convincing after adjusting for sociodemographic characteristics, lifestyle factors, and health conditions, as all these factors can affect the relationship between self-reported sleep duration and VI.

In fact, the potential impact between sleep and vision is underestimated. A health care and rehabilitation system that covers vision and sleep must be established and popularized. The system should take into account the possibility of VI in subjects with abnormal sleep duration as well as abnormal sleep duration in subjects with VI and engage in effective management accordingly. As for subjects with abnormal sleep duration, their vision function should be taken care of and examined regularly. As for subjects with VI, their sleep-wake cycles should be given close attention. Sleep medication, physical therapy, or psychotherapy treating sleep difficulties may be helpful in reducing the incidence of VI. Vision impairment medication or surgery may contribute to setting up circadian rhythm and improving abnormal sleep duration (42, 43).

The present study is not without limitations, which can be summarized as follows:

1. Due to the cross-sectional nature of the study, causality could not be established. Prospective cohort studies may need to clarify the causality between sleep duration and VI.
2. Sleep duration and visual function were subjectively assessed through self-reported questionnaires. If an objective assessment of sleep quality had been obtained using polysomnography or actigraphy, and vision had been tested by visual acuity chart, the results would be more robust. However, self-reported questionnaires of sleep duration and visual function are widely accepted in a number of large-scale population-based studies, and self-reported sleep duration is sufficiently consistent with objective methods, such as actigraphy (44, 45).
3. Self-reported sleep duration could not allow for the differentiation between total sleep time and time in bed. However, previous studies have shown that objective measures of total sleep time and time in bed were highly correlated (7, 46).
4. The correlation between the severity of VI and the severity of sleep difficulties was not explained. There was a lack of data about the severity of VI and the severity of sleep difficulties in the CHARLS survey. Future studies may need to address this problem.
5. The study was not adjusted for mood, medications, or exposure to artificial light at night, which may affect sleep duration (47, 48). They were not taken into account in previous studies (37, 48). However, we found the relationship was maintained throughout all three models after adjusting for sociodemographic data, lifestyle factors, and health conditions. Future studies may need to take more factors into account.

## Conclusions

In summary, based on large-scale population-based data in China, both short and long sleep durations showed an association with VI. However, the cause-and-effect relationship is unclear. The relationship of VI to sleep in the elderly requires more attention because of a lack of epidemiological studies in China. A more comprehensive and integrated healthcare and rehabilitation system that addresses vision and sleep is also needed.

## Data Availability Statement

The raw data supporting the conclusions of this article will be made available by the authors, without undue reservation.

## Author Contributions

MS, QB, and MZ performed material preparation, data collection, and analysis. MS wrote the first draft of the manuscript and all authors commented on previous versions of the manuscript. All authors contributed to the study conception and design. All authors read and approved the final manuscript.

## Funding

This research was supported by the Shanghai Natural Science Foundation (19ZR1440900), Shanghai Pujiang Program (2019PJD047), and National Natural Science Foundation of China (81500714).

## Conflict of Interest

The authors declare that the research was conducted in the absence of any commercial or financial relationships that could be construed as a potential conflict of interest. The reviewer BJ declared a shared affiliation with several of the authors QB, BL, XS, and MZ to the handling editor at time of review.

## Publisher's Note

All claims expressed in this article are solely those of the authors and do not necessarily represent those of their affiliated organizations, or those of the publisher, the editors and the reviewers. Any product that may be evaluated in this article, or claim that may be made by its manufacturer, is not guaranteed or endorsed by the publisher.

## Abbreviations

VI: visual impairment
OR: odds ratio
CI: confidence interval
CHARLS: China Health and Retirement Longitudinal Study.
